# Single–Molecule Biodosimetry

**DOI:** 10.1021/acs.analchem.5c03303

**Published:** 2025-08-20

**Authors:** Michael Lamontagne, Shannon M. Newell, Ileana M. Pazos, Ronald E. Tosh, Jerimy C. Polf, Michael Zwolak, Joseph W. F. Robertson

**Affiliations:** † Biophysical and Biomedical Measurement Group, Microsystems and Nanotechnology Division, Physical Measurement Laboratory, 10833 National Institute of Standards and Technology, Gaithersburg, Maryland 20899-3460, United States; ‡ Dosimetry Group, Radiation Physics Division, Physical Measurement Laboratory, National Institute of Standards and Technology, Gaithersburg, Maryland 20899-3460, United States; § M3D, Inc. Ann Arbor, Michigan 48104, United States

## Abstract

Inferring characteristics of radiation exposure using biological molecules is extremely challenging. Current methods, in particular, lack a clear connection between dose and molecular response. Here, we demonstrate that resistive–pulse nanopore sensors enable single–molecule biodosimetry by quantifying the frequency of double–strand DNA scissions versus γ radiation dose. The resulting response curve shows an elongated Gaussian behavior, reminiscent of cell survival rates versus dose. We demonstrate that the competition of radical damage of DNA—i.e., single–strand lesions that lead to breakage—with bimolecular radical loss captures the form of the response. Our sensors and protocol provide a foundation for numerous technological advances. These include rapid dosimetry for triage in emergency situations and ex vivo monitoring of radiotherapy effectiveness in order to tailor treatment to patient– and tumor–specific response.

## Graphical abstract

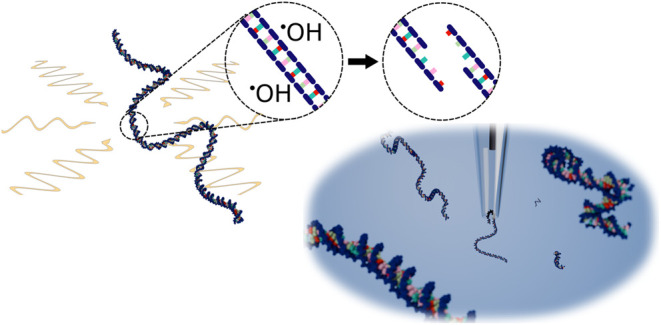

## Introduction

Understanding the biological consequences and damage mechanisms of ionizing radiation is central to cancer therapy.^1^ Quantifying radiation exposure, in particular, enables post–therapy assessment of the delivered dose^2^ and will be important in other scenarios, such as radiological accidents^3^ or nuclear conflicts.^4^ Moreover, beyond the acute effects of high–dose exposure (>2 Gy), the accumulated impact of low–dose exposure (≈100 μGy), such as from medical imaging or working in mines, is a poorly understood factor in assessing health.^5^ Current tools are often inadequate for these medical and emergency applications.^6^ For instance, the “gold standard” for retrospective dosimetry—dicentric chromosomal analysis (DCA)—requires >48 h of preparation time for cell culturing after sample collection to produce actionable data.^7^ Furthermore, DCA only produces valid results at exposures <5 Gy.^8,9^ Other chromosomal counting assays and proteomic tools have similar issues. Leveraging advances in biotechnology and nanoscience is a promising route to overcome such challenges and provide tools to quantitatively assess damage and mechanisms, as well as to rapidly and accurately measure the exposure of individuals under a broad range of conditions.^5^

In this regard, the most well–studied biomarker for assessing radiation damage is DNA. It has long been known that radiation–induced damage to genomic DNA can lead to cell death,^10,11^ with as little as one double–strand break (DSB) being sufficient in certain circumstances.^12^ In living systems, cell death can be mitigated by the action of DNA repair enzymes.^13,14^ However, in a buffer containing DNA, in addition to the lack of molecular packing (as in cells),^15^ radiation damage proceeds unperturbed by repair enzymes. This yields a more straightforward picture of the damage mechanisms and rates. For DNA, damage includes both *direct* and *indirect* lesions on the bases or sugars. In practice, irradiation deposits the most energy in water—the most abundant species by mass. This initiates a cascade of reactive hydrolysis products, including ^•^OH, e_aq_^–^, H^•^, HO_2_^•–^, O_2_^•–^, H_3_O^+^, H_2_, and H_2_O_2_.^16^ Of these, ^•^OH, e_aq_^–^,O_2_^•–^ and H_2_O_2_ are particularly damaging to DNA, with ^•^OH specifically associated with strand scission.^17–19^ The predominant mechanism for strand–breaking reactions begins with H–abstraction on the ribose, which occurs in 10 to 20% of all ^•^OH reactions with DNA, with ≈20% of these abstraction reactions leading to strand–break reactions.^20,21^

Nanopore sensors emerged as a tool to study the properties of DNA and other biopolymers in 1996.^22^ The core sensing principle is similar to other biophysical research tools, such as the Coulter counter:^23,24^ A thin membrane (with a narrow pore) partitions an electrolyte solution, where a voltage drop drives an ionic current from one side to the other. When the analyte is driven through the pore—via fluid pressure for a Coulter counter or electrophoretically for DNA—a resistive pulse occurs in the current. The capture process itself is complex. The electric fields and polymer dynamics,^25,26^ as well as the interplay between electrophoretic and electroosmotic forces with the effective charge of the molecule,^27,28^ all play a role. The molecular volumes of nanopores (rather than cellular volumes of Coulter counters) allow the system to measure single molecules. In particular, the magnitude of the resistive pulse is proportional to the volume of the pore occupied by the translocating molecule.^29,30^ For long, uniformly–charged molecules, such as DNA, integrating the ionic current pulse (against time) yields the net charge of ions which were excluded by the DNA, known as the equivalent charge deficit (ECD) a value proportional to the molecular length.^31,32^

Since their introduction in 2001,^33^ solid–state nanopores (ssNP) have become a workhorse of single–biomolecule sensing.^34^ The most common approach is to fabricate nanopores in ultra–thin silicon nitride membranes, which enables sampling above megahertz frequencies and therefore maximizes discrimination of sublevel blockades within individual translocations.^35,36^ While these pores are highly susceptible to fouling, particularly in media that contains proteins or single–stranded DNA, this can be mitigated through advances in surface–modification.^37–39^ Other geometries and materials, such as oxide–based nanopores^40–42^ or conical pores, can also improve the measurement.^43,44^ In this work, we employ on–demand laser–pulled capillaries,^45,46^ which serve as a simple, deployable alternative to more common membrane based nanopores.

Yet, like all ssNPs, nanocapillaries show significant pore–to–pore variability in both geometry and surface chemistry. Additionally, DNA capture by a nanopore is a complicated physical process that is dependent upon molecular length^47–49^ and can be affected by the ratio of electrophoretic and electroosmotic flow through the pore.^27^ We employ pulled quartz nanopipettes to demonstrate single–molecule biodosimetry, significantly improving measurement quality over fluorescent measurement systems like gel electrophoresis.^50,51^ We further provide a method that enables precision measurement in systems with large pore–to–pore variation and will support quantitative measurements of DNA damage across laboratories to enable inter–laboratory comparisons and standardization.

## Methods

### Sample Preparation

The buffer of as–received 2.5 kbp DNA (Thermo Fisher, NoLimits) was exchanged from 1× TE (10 mM tris, 2 mM EDTA) to 20 mM sodium phosphate (pH = 7.4) using 1 kDa cut–off mini dialysis tubes (Cytiva) to minimize the radical scavenging properties of tris, and the DNA was diluted to 10 nM for irradiation. DNA concentrations were confirmed at all points via ultraviolet (UV) measurement with a NanoDrop spectrophotometer (Fisher Scientific).[Fn fn1] The DNA containing buffer was transferred in 300 μL aliquots into 0.5 mL DNA LoBind tubes (Eppendorf, Cat. no. 022431005). Each solution was then exposed to a γ–ray field from a Gammacell 220 ^60^Co irradiator (Nordion, Canada) for the time required to achieve the desired dose. The dose rate was 1.373 Gy/min and the irradiation temperature was 23 °C. The uncertainty in absorbed dose in water is bounded by ±1.6% for this geometry. Postirradiation, a measurement solution was prepared by diluting the DNA to 3 nM, adding internal standards of 5 kbp and 10 kbp at 0.3 nM DNA into an electrolyte buffer with a final concentration of 4 M LiCl 1× TE.

### Pipette Characterization

Quartz nanopipettes were prepared from capillaries (1 mm O.D., 0.5 mm I.D.) with a laser assisted glass capillary puller (P-2000 Sutter Instruments, Novato, CA). Parameters such as the laser power, delay time, and strength of pull were adjusted to optimize the terminal diameter and interior geometry of the pipettes to maximize the measurement resolution of a particular analyte molecule or range of molecules. In this case, pipettes were optimized to achieve an acceptable success ratio in clearly defined log­(ECD) frequency peaks for molecules in the 2.5 kbp to 10 kbp size range. The laser puller program^52^ used was as follows: HEAT = 575, VELOCITY = 25, DELAY = 180, PULL = 225.

Nanopipettes were filled with a solution of 4 M LiCl and 10 mM TE buffer at pH 7.4. The nanopipette was immersed into the DNA-containing buffer. The measured concentration of irradiated DNA is calculated by comparing the average area of the 5 and 10 kbp peaks in counts, to the known concentration at which they were added. The integrated area of the 2.5 kbp peak is taken as the measured concentration of intact DNA for each dose. Each measurement was performed by a separate nanopipette to avoid cross contamination. A +700 mV potential difference was applied between the ground (exterior of pipet) and sensing (interior of pipet) electrodes, and ionic current was monitored with an Axopatch 200B amplifier (Molecular Devices, San Jose, CA) sampling at 500 kHz, with a lowpass Bessel filter of 10 kHz. The ionic current data was analyzed using a threshold algorithm set at 5σ, where σ is the standard deviation of the ionic current to identify DNA–based resistive pulses. The signals were then decoded with a modified CUSUM-based algorithm^53^ as implemented in the Nanolyzer software package (Northern Nanopore, Ottawa, Canada).

## Results and Discussion

The single–molecule biodosimetry approach we develop employs nanopores to quantify double–strand breaks from an irradiated sample. Here, we expose a series of aqueous solutions containing 2.5 kbp DNA, Figure 1a, to γ radiation with doses up to 15 Gy, which results in double–strand breaks, Figure 1b. We note that the total DNA concentration is unaffected by radiation as judged by UV absorbance at 260 nm/280 nm, see Figure S1 of the Supporting Information (SI). The variance in the terminal diameter and conductance of the capillaries after fabrication (see Figure S2 (SI)) is significant enough to complicate the analysis of immediate trends in translocation data recorded in different capillaries. In order to extract quantitative information and compare data between different capillaries, two longer DNA molecules (here, 5 and 10 kbp) are added post–irradiation as simultaneous concentration and molecular–length standards.

**Figure 1:**
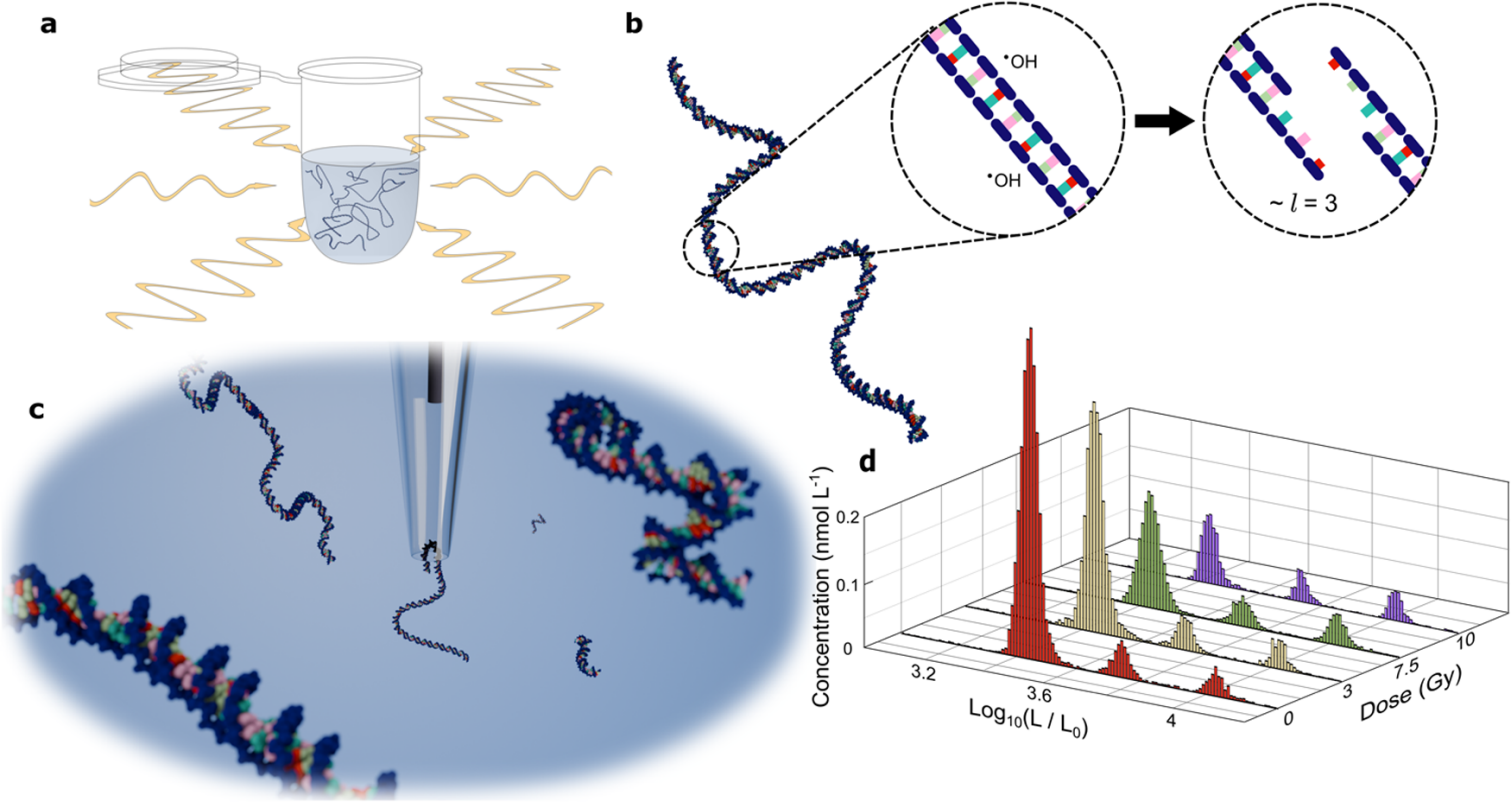
Nanopore-based single-molecule dosimetry. (a) An aqueous solution of 2.5 kbp DNA is irradiated with a ^60^Co calibrated γ source. (b) Single–strand breaks in the sugar–phosphate backbone accumulate until two breaks occur close enough (here shown as *l* = 3 bases) to compromise the stability of the molecule and cause a double–strand break. (c) Irradiated DNA sample is quantified post–exposure with a glass nanopipette. (d), Histograms of the concentrations of the irradiated 2.5 kbp and unirradiated internal standards of 5 kbp and 10 kbp DNA as a function of dose (*L*_0_ = 1 bp). Absolute size and concentration are calculated from the ECD and capture frequencies by calibrating against these two internal standards.

During measurement, DNA is electrophoretically driven into the mouth of a glass nanopipette, Figure 1c. The resulting current blockades—i.e., the resistive pulses—are analyzed and compiled into a histogram of molecular length estimated from the ECD,^31^ Figure 1d. Figure 2 shows raw current versus time data from two separate capillaries demonstrating the variation that we observe from trial to trial. In Figure 2a, the current pulses are about 100 to 200 pA, while Figure 2b shows current pulses roughly twice that magnitude. This effect is not connected to the radiation dose to the molecule, see Figure S3 in the SI for the full data set, and the trend is related to uncontrolled differences in the geometry and surface chemistry of the capillaries. Due to issues of cross contamination, and nanopipette fouling, it is not feasible to use the same pore for every dose. These common limitations for solid–state nanopores require methods to account for pore–to–pore variations even among a series of identically prepared capillaries. Building on prior work that calibrated the concentration using the capture frequency of an internal standard,^54^ we introduce two internal standards, which provides a correction for both size and concentration simultaneously. Quantitative analysis of these data is obtained by integrating the resistive pulses (shaded in Figure 2a,b) and constructing a histogram of the ECD (Figure 2c). These histograms highlight the shifts that arise from capillary effects. Aligning both ECD and counts produces quantitative histograms of concentration versus length (Figure 2d). Moreover, the introduction of two standards improves the uncertainty quantification and helps identify potential systematic biases (e.g., arising from size–dependent capture rates^47,48^ ).

**Figure 2:**
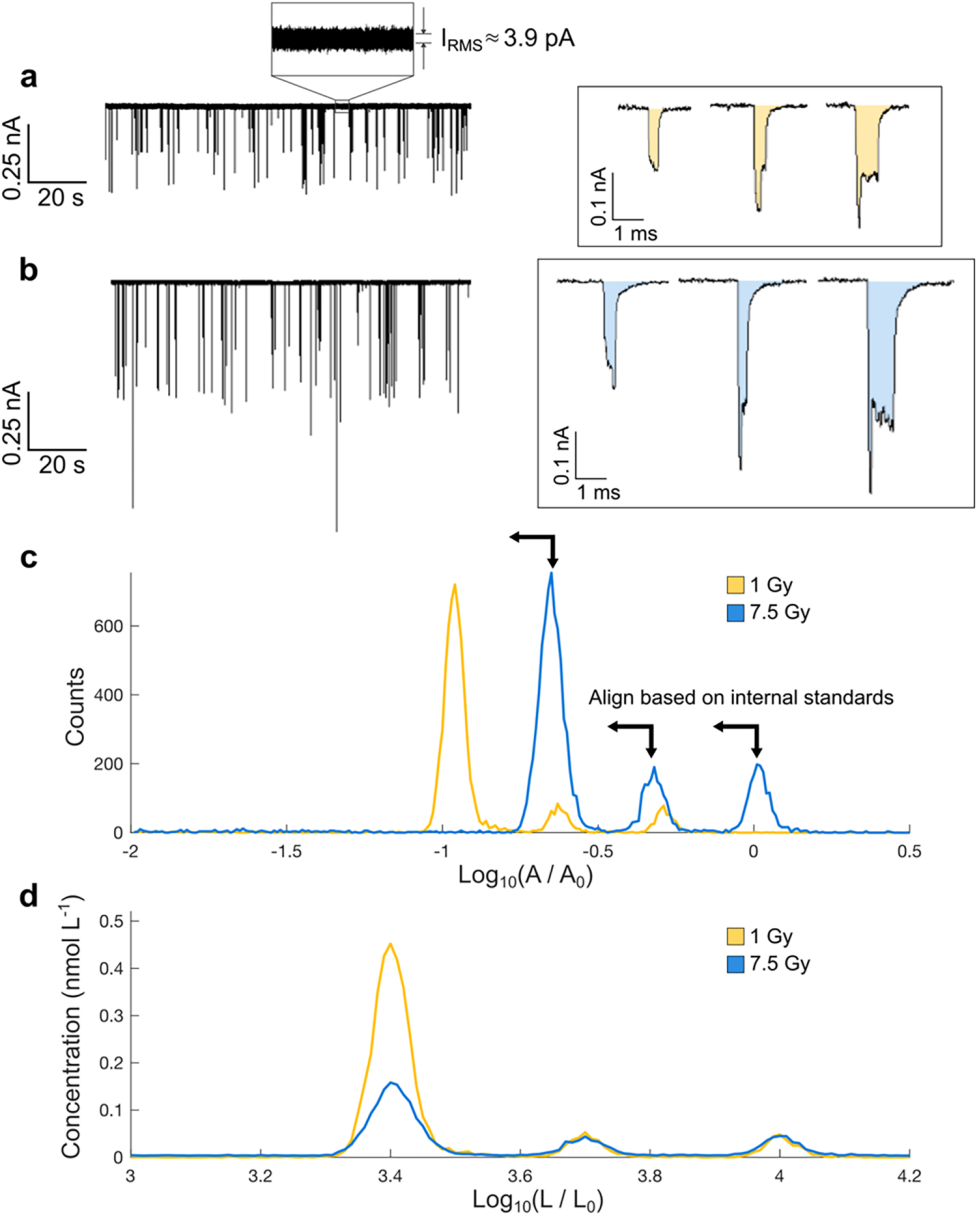
Molecular standards as an internal calibration and ruler. (a) Ionic current versus time showing resistive pulses of the 5 and 10 kbp DNA internal standards and 1.0 Gy γ irradiated 2.5 kbp DNA. (b) Ionic current of an identical DNA mixture in which the 2.5 kbp DNA has been irradiated at 15.0 Gy. Characteristic current events for the irradiated length, 2.5 kbp (left), and the two internal standards, 5 kbp (center) and 10 kbp (right), are shown for 1.0 and 7.5 Gy enlarged in the boxes. The ECD is the area, A, shaded in orange or blue. (c) Log­(ECD) histograms for the 1 and 7.5 Gy samples without calibration (here, *A*_0_ = 1 pC). (d) The same data sets after calibration against the internal standard peaks (here, *L*_0_ = 1 bp). After calibration, the final 1.0 and 7.5 Gy histograms show a decreasing concentration of intact 2.5 kbp DNA with dose.

The resulting dose–response curve, Figure 3, agrees well with the following stochastic model. A double–strand break requires two nearby single–strand lesions on opposite strands. For any nucleotide, a single–strand lesion occurs with probability *P*_Λ_. A lesion on one strand can combine with a lesion on the other strand anywhere within *l* bases, resulting in a double–strand break with two dangling single–stranded ends of length *l*. For example, *l* = 0 means the lesions have to be at the same location along the duplex. Effectively, *l* plays the role of a stability parameter, within this distance two lesions on opposite strands destabilize the duplex. At each nucleotide in DNA, except near the strand ends, the probability that a double–strand break occurs is thus

1 $$P_{\Lambda }\cdot \left(2l+1\right)P_{\Lambda }$$

The probability that the whole double–strand of DNA remains intact, i.e., that there are no double–strand breaks, is

$$P=(1-P_{\Lambda }\cdot \left(2l+1\right)P_{\Lambda }{)}^{L}\approx \exp(-\mathrm{L̃}P_{\Lambda }^{2})$$ 2

where *L̃* = (2*l* + 1)*L* is an effective length of the DNA in number of base pairs that captures how many ways the DNA can break. This model is consistent with the classic model of Freifelder and Trumbo,^55^ as well as that of Thomas,^56^ but includes the SSB distribution. Their data validates the model form and thus it should hold for varying DNA concentrations and lengths. We note that this expression neglects end effects, which is justified so long as *l* ≪ *L*. We will see that this is the case. The approximate Gaussian form for small *P*_Λ_ upper bounds the expression on the left-hand side for all *P*_Λ_, which is important for interpreting the results.

**Figure 3:**
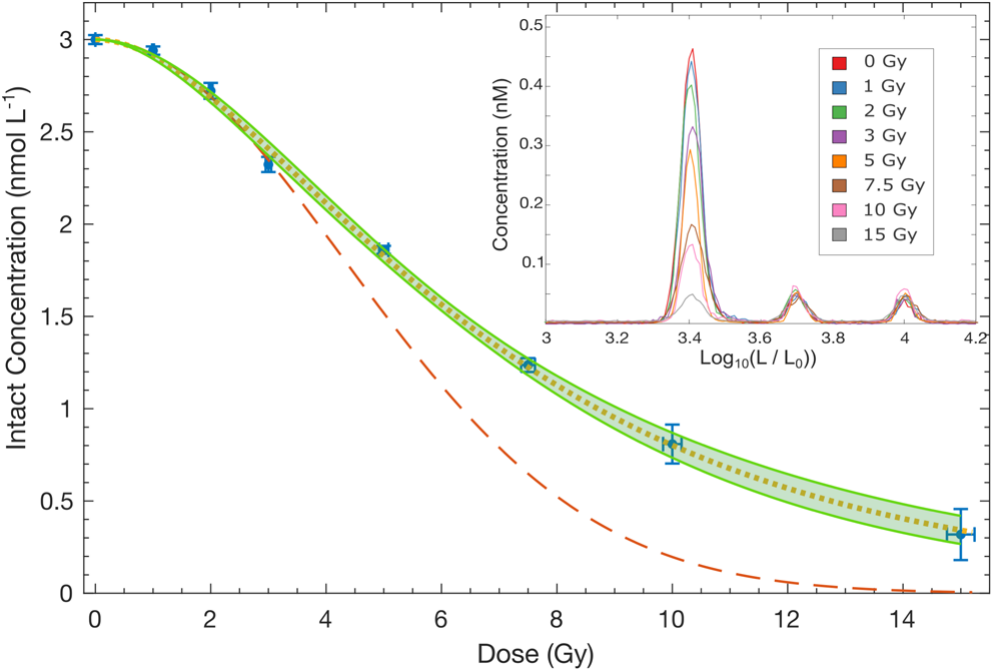
DNA dose response. The intact DNA concentration (blue circles and error bars) versus dose shows that the response is roughly Gaussian. At very low dose, one expects such behavior since bimolecular radical decay will be negligible. However, the best fit (red, dashed line) at low dose, ≤3 Gy, to a Gaussian model does not work well at high doses, overestimating the loss of intact DNA. A best fit to the whole range of data (not shown) similarly does not fit well (overestimating intact DNA at intermediate doses and underestimating at high doses), as the shape of the curve is not actually Gaussian in *D* but an elongated Gaussian. Including bimolecular decay yields a two parameter model and a high–quality fit to the data (orange, dotted line). The inset shows the measured concentration of three key molecules. The concentration of 2.5 kbp DNA decreases with increasing dose. Error bars are a scaled average of the standard deviation between the measured concentrations of the 5 and 10 kbp internal standards (that were fixed between runs). The scaling is based on the length of the data set and the number of 2.5 kbp events recorded at a particular dose.

There are only two variables in eq 2: The lesion probability, *P*_Λ_, and the stability length *l*. Thus, regardless of whether the lesions originate from direct radiation damage or indirectly from reaction of the DNA with hydrolytically–produced radicals, they will deplete the initial intact DNA concentration Φ_0_ according to a universal form in the small *P*_Λ_ limit

$$\Phi =\Phi_{0}\exp(-\mathrm{L̃}P_{\Lambda }^{2})$$ 3

While eq 3 is general, we consider only indirect damage via radical reactions, as the mass fraction of DNA is very small in solution.

To lowest order in dose, *D*, the probability to generate a lesion should grow linearly, i.e., *P*_Λ_ ∼ *D*. A reaction model where radicals damage nucleotides and are also subject to (effective) unimolecular decay gives such a linear dependence provided the initial radical concentration formed by water radiolysis itself is linearly dependent on *D*. This linear dependence would give a Gaussian decay versus dose, Φ = Φ_0_ e^–κ*D* ^2^^, with κ the decay coefficient. Figure 3 is reminiscent of Gaussian decay of Φ with *D*. Yet, a low–dose (≤3 Gy) fit (the red, dotted line in Figure 3), where this model should be valid, gives significantly less intact DNA than expected at large *D*. As we note above, the approximation in eq 2 upper bounds the exact expression, and thus it can not be responsible for this deviation. Therefore, another important reaction or other process must be present.

Bimolecular decay of radicals, i.e., two radicals combine to form an inert species, may be such a reaction.^16^ We thus consider the set of reactions

4 $$N+R\overset{k_{b}\;}{\to }\Lambda$$

5 $$R\overset{k_{\alpha }\;}{\to }⌀$$

6 $$R+R\overset{k_{\beta }\;}{\to }⌀$$

where *N* is the total nucleotide concentration, *R* the radical concentration (primarily ^•^OH), and Λ the total defect concentration. Defects occur via a bimolecular reaction of nucleotides and radicals with rate *k*_b_. Radicals decay via a unimolecular process with rate *k*_α_ and a bimolecular process with rate *k*_β_, both resulting in annihilation of the radicals to an inert chemical species, ⌀. All reactions are taken to be irreversible since they involve a substantial energy dissipation.

The initial total nucleotide concentration during irradiation is *N*_0_ = 2*L*Π_0_, where *L* is the length of the DNA and Π_0_ is its initial concentration of DNA during irradiation (Π_0_ = 10 nM). The initial radical concentration is taken to be linearly proportional to the radiation dose according to the well–known *G*–value^57^

$$R_{0}=G\cdot D$$ 7

The constant of proportionality, *G*, is the radiation chemical yield.^58^ We assume that immediately after γ irradiation the solution contains a uniform distribution of hydroxyl radicals and all nucleotides are equally likely to be damaged. Finally, the initial defect concentration is zero, Λ_0_ = 0.

In the limit of *N*_0_ ≫ Λ, we can analytically solve the reaction equations for the set in eq 4. After all radicals react, the defect probability is

8 $$P_{\Lambda }=\frac{\Lambda \left(t\to \infty \right)}{N_{0}}=\frac{k_{b}}{2k_{\beta }}\log\left[1+\frac{2k_{\beta }R_{0}}{k_{b}N_{0}+k_{\alpha }}\right]$$

If this bimolecular loss mechanism is present, the intact DNA still follows eq 3 but with a defect probability given by eq 8. The defect concentration, Λ­(*t* → ∞) will be zero when *N*_0_ is zero, even though the probability, eq 8, remains finite. The model has several parameters. Yet, as a fit to the experimental data here, it has only two effective parameters

9 $$K^{2}=\mathrm{L̃}{\left(\frac{k_{b}}{2k_{\beta }}\right)}^{2},\Gamma =\frac{2k_{\beta }G}{k_{b}N_{0}+k_{\alpha }}$$

With *K* and Γ, the experimental data is fit extraordinarily well (green, dashed line in Figure 3).

We note that these two parameters are correlated, with a decrease in one generally occurring with an increase in the other (i.e., *K*·Γ is roughly constant since the initial decay is Gaussian). The best fit to the data (without accounting for uncertainty) is *K* = 1.23 and Γ = 0.155 Gy^–1^. Including uncertainty, the fit is presented in Figure 3. The orange dotted line is the average intact concentration from the joint distribution of K and Γ, and the shaded region given by plus and minus one standard deviation. Treating the two parameters independently gives *K* = 1.3 ± 0.4 and Γ = (0.16 ± 0.05) Gy^–1^. This does not capture their correlation. Yet, their correlation ensures that the low–dose decay is correct, as well as lowers the error.

Extraction of any of the fundamental parameters in eq 9 (i.e., *L̃*, *k*_*b*_, *k*_α_, *k*_β_, or *G*) requires either tuning various quantities (e.g., concentrations) or independently measuring or estimating a subset of those parameters. For instance, the ratio of *k*_*b*_ (single–strand break rate) to *k*_β_ (bimolecular radical decay) depends only on *L̃* and K, see eq 9. Prior measurements provide the length scale, *l*, for which single–strand breaks on opposite strands lead to a DSB, which is linearly related to *L̃* via *L̃* = (2*l* + 1)*L*. Those measurements give *l* from about 3 bp to 16 bp for high and low ionic strengths, respectively,^55^ or *L̃* between 3*L* and 31*L*. Taking *L̃* = 10*L*, inline with the conditions here, gives *k*_*b*_/*k*_β_ = 0.017 ± 0.005.

The value of *G* allows us to go one step further and quantify *k*_α_ (unimolecular radical decay). Prior measurements of the *G* for ^•^OH give, in conventional units, 2.56 to 5.5 molecules per 100 eV of deposited energy.^16,59^ Taking *G* = 2.56, which is relevant for the conditions here, gives *k*_α_ = (3.4 ± 0.4) *k*_*b*_ *N*_0_. The radical scavenging rate is thus of the same order as *k*_*b*_ *N*_0_, the total single–strand break rate. This suggests that the dominant (effective) unimolecular decay route is to create DNA damage that does not lead to breakage. Future experiments (e.g., versus *N*_0_ and *L*) can unambiguously demonstrate this molecular mechanism.

From these data and the chemical model, we can estimate the limit of detection (LOD) and uncertainty for absorbed dose. Using the elongated Gaussian, eq 3, and the average uncertainty in the measured intact DNA concentration, we estimate a lower LOD of ≈0.8 Gy at a 2σ threshold and a measurement resolution of <0.3 Gy for absorbed dose <6 Gy. Reducing the LOD to below ≈0.8 Gy will require other strategies. One approach would to quantify base lesions, which occur at roughly 100× the frequency of strand scissions, through direct observation,^60^ nanopore sequencing,^61^ or through enzyme–directed labeling strategies.^62^ Including base damage detection could extend the effective range of these sensors from 0.8 mGy to >15 Gy, which covers much of the measurement space for human health.

As just discussed, we measured a universal, reproducible trend with multiple solid–state nanopores. This has generally proven elusive, even within pores that are nominally identical, for a number of reasons. Pore–to–pore variability in diameter, internal taper, and surface charge and roughness, among other factors, has a significant impact on characteristic molecular signatures, such as ECD. Details can be found in the Supporting Information section. We have taken a two–pronged approach to overcome the challenges in getting quantitative trends. First, we employ relatively large nanopipettes (i.e., ≈12 to 17 nm), which leads to a number of advantages over reliance upon smaller pores in addition to ease of use: (i) Large pores frequently enable collection of large data sets (approximately 10 k events before pore fouling); (ii) They are more robust, filling the narrow sensing region of the tip completely a larger percentage of the time and giving a higher yield of pores with very low baseline noise (*i*_RMS_ ≈ 4.4 pA); and (iii) They have a flat capture probability across the size range of the three target molecules as shown in Figure 4c. Incidentally, the nanopipettes also make sample recovery trivial, as the analyte is measured in the same vial in which it is prepared as opposed to a flow–through system in which contamination, dilution, and other complications can arise. Second, we correct for the analyte size and capture–rate fluctuations that result from the natural pore–to–pore variability by using the fixed sizes and concentrations of the two internal molecular standards, which allows us to infer the concentration of all molecules in our target size range, from 2.5 to 10 kbp, for our nanopipettes.

**Figure 4:**
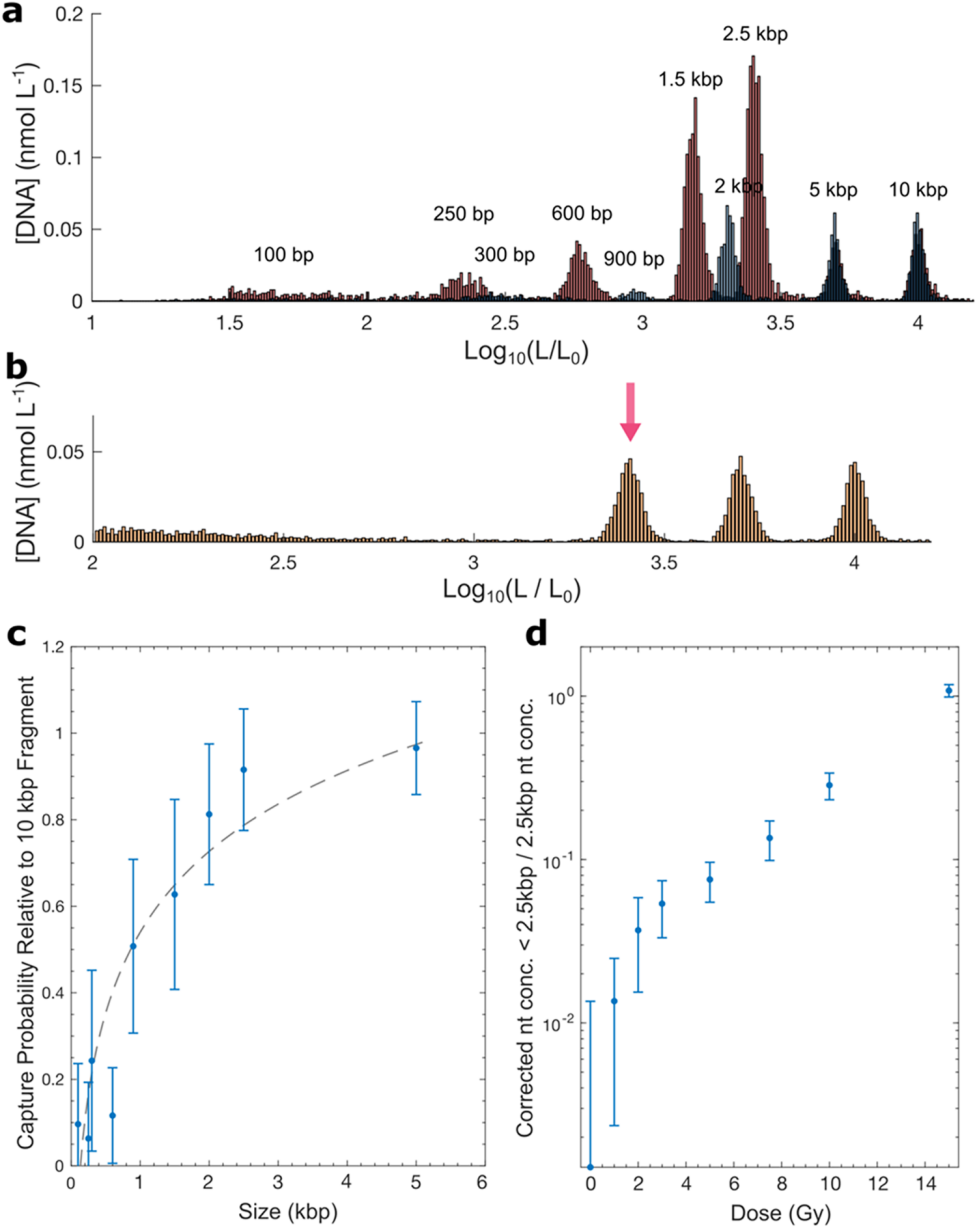
Fragment concentration bias. (a) A comprehensive molecular ladder of ≤10 kbp DNA run through a pipet prepared identically to those used in the primary experiments. Separate ladders containing 100, 250, 600 bp, 1.5, 2.5, 5, and 10 kbp fragments (red), and 300, 900 bp, 2, 5, and 10 kbp fragments (blue) were recorded and aligned as described in the Methods to minimize overlap. (b) Molecular size distribution for all captured molecules after exposure to 15.0 Gy of radiation. Fragments between 100 bp and 2.5 kbp are visible below the 2.5 kbp peak indicated with the red arrow (*L*_0_ = 1 bp). (c) The probability, *P*_*C*_, of successfully capturing a DNA fragment of a given size as calculated from the ladder shown in (a). Error bars are the standard deviation of the best–fit parameters of a logarithmic fit (black, dotted line) to the capture rates of the ladder run through three identically prepared pipettes. (d) The ratio of nucleotides in fragments (the difference between a “fragment” and the intact Gaussian fitted peak being >2 standard deviations) to nucleotides in the intact 2.5 kbp DNA after correcting for the deflated *P*_*C*_ of small molecules.

This approach enables low uncertainty, molecule–by–molecule measurement of the intact DNA concentration. Yet, using larger pores and optimizing the standards around them is not without trade off: We lose resolution of the concentration of small fragments. This is further compounded by the use of 4 M LiCl as the buffer and 700 mV as the applied voltage, As previously observed by Wanunu et al.,^47,48^ and discussed in detail in Bell et al.,^49^ at these conditions the capture rate is highly length dependent. However, there is a compromise between the length dependence of the capture rate and maximal resolution of molecular size. At high salt concentrations there is increased Manning condensation of counterions along the DNA backbone,^63^ reducing the effective DNA charge, and causing the DNA molecules to translocate more slowly, yielding higher resolution of the log­(ECD) peaks. Additionally, the pores are less likely to foul with increasing voltage, leading to larger data sets at higher rather than lower voltages. In these experiments, peak resolution was chosen as the higher priority to optimize the accuracy in the measured concentration of the target size (2.5 kbp). This compromise improves our ability to fit the dose–dependent data using eq 3 in Figure 3 at the expense of detection and characterization of smaller fragments.

Despite this compromise, we are still able to quantify smaller fragments with sufficient precision to estimate ionizing radiation effects. Figure 4a shows the concentration across a wide range of molecular sizes, where the weight on the smaller fragments is lower than expected. There are two primary reasons for this. For small molecules, it is not possible to fully distinguish capture and translocation, partial capture, and system noise. Also, there is a complicated interplay of physical processes on the capture of molecules diffusing near the terminal aperture. Generally speaking, for negatively charged surfaces, the electro–osmotic force on the exterior of the tip of the nanopipette, mediated through fluidic drag interactions, creates a spherical region of pre–concentrated DNA around the pore mouth. At the pipet tip, there is a switch in regimes where the electrophoretic force becomes non-negligible. Molecules pre–concentrated near the tip fluctuate in and out of the high electric field immediately inside the vestibule of the nanopipette, with a length dependent free energy barrier, ultimately giving single–file capture. These three factors: the electrophoretic force, the free energy penalty for confinement within the nanopipette tip, and diffusion of the DNA lead to a lower number of successful small molecule (relative to the pore) translocations than their concentrations would indicate, with a preference that increases with applied voltage.^25,27^

In other words, the correction of capture events needs to be non–linear. DNA standards with a size of the same order as our target DNA permits a linear correction for the larger size molecules but under weights small fragment events. As a preliminary estimate, we measure the bias, Figure 4c, of a ladder of known fragment concentrations below the intact DNA size. This is done with three nanopipettes identically prepared to those in the primary experiments. We note that this provides an average correction factor rather than a pore–by–pore calibration as was done for the primary experiments. Consequently, the error bars are larger for the small fragments. Despite this lack of optimization, the corrected ratio of measured DNA fragments (in nucleotides) to intact 2.5 kbp DNA (in nucleotides) monotonically increases with dose, see Figure 4d. In some scenarios, such as emergency exposure, the initial concentration of undamaged DNA in a sample will not be known. Under such conditions, a ratiometric approach—i.e., measuring the concentration of fragments relative to intact DNA—may enable determination of the dose to the needed accuracy by employing ratio–dose response data like that in Figure 4d. We anticipate that ratiometric data can be significantly improved by application of a quantitative model for length dependent capture such as in Bell et al.,^49^ or by operating under conditions where the capture rate is not sensitive to the length of the molecule.^25^

## Conclusions

We demonstrate single–molecule biodosimetry using quartz nanopipettes. Calibration against internal molecular standards, as we introduced here as a parallel to DNA electrophoresis ladders, is essential for the acquisition of fully quantitative results and will be critical to the adoption of nanopores in applications outside of specialized academic laboratories. The DNA dose–response curve exhibits an elongated Gaussian shape—one similar to cell death response curves. This strongly indicates double–strand DNA breakage proceeds with three key characteristics in the relevant dose range for clinical and emergency response applications: (i) indirect single–strand breakage by reaction with radicals, (ii) nearby single–strand breaks on opposing strands generate a DSB, and (iii) damage competes with bimolecular radical decay. This agrees with prior results that suggest reaction with radicals, rather than direct damage with high–energy particles, is the dominant mechanism of DNA damage in near physiological conditions.

Nanopore–based sensors thus show promise for a wide range of applications, including rapid dosimetry after accidental, imaging, or therapeutic radiation exposures. Here, we demonstrate <0.3 Gy precision for dosimetry between ≈1 and 6 Gy with quantification >15 Gy, a critical dose range for both retrospective dosimetry and radiation oncology that current tools do not address. We project the total analysis time with an engineered device can be made with minimal sample processing and be done in less than 1 h. Additionally, DNA damage due to different types of external beam radiation (x–rays, electrons, protons, etc.) or radiopharmaceuticals that emit many types of short-range radiation (e.g., soft x–rays, auger electrons, α particles, β particles), could be measured and characterized. We believe this data would provide quantitative information on the sensitivity of DNA to a range of different energies, linear energy transfer, dose rates, and other properties of radiation. Such information would be a fundamental building block to new models and dose calculations that improve how we use radiation to treat cancer.

## Supplementary Material



## Footnotes

1 Certain commercial materials, equipment, and instruments may be identified in this work to describe the experiments as completely as possible. In no case does such an identification imply a recommendation or endorsement by the National Institute of Standards and Technology, nor does it imply that the materials, equipment, or instrument identified are necessarily the best available for the purpose.

## Notes

Details on the laser pulling process and settings, aperture size and variance of the nanopipettes, as-irradiated DNA concentration, and raw nanopore signals and data extraction tools (PDF)
