# Supplementary material for: Single–Molecule Biodosimetry
Source: Anal Chem. 2025 Aug 20;97(40):22004–12. doi: 10.1021/acs.analchem.5c03303 (PMC12529473; doi:10.1021/acs.analchem.5c03303)
Supplement: Supplementary file 1 [file ac5c03303_si_001.pdf]

Supporting Information: Single–Molecule Biodosimetry

Michael Lamontagne,[†] Shannon M. Newell,[†] Ileana M. Pazos,[‡] Ronald E. Tosh,[‡] Jerimy C. Polf,[¶] Michael Zwolak,[†] and Joseph W. F. Robertson[*,†]

†*Biophysical and Biomedical Measurement Group, Microsystems and Nanotechnology Division, Physical Measurement Laboratory, National Institute of Standards and Technology, Gaithersburg, MD, USA*

‡*Dosimetry Group, Radiation Physics Division, Physical Measurement Laboratory, National Institute of Standards and Technology, Gaithersburg, MD, USA*

¶*M3D, Inc. Ann Arbor, MI 48108*

E-mail: joseph.robertson@nist.gov

Contents

Laser pulling process **S-2**

Sample validation **S-3**

Pipette characterization **S-4**

Ionic current signals **S-5**

In this supplemental information, we provide additional details for the laser pulling process and settings, DNA concentration, raw nanopore signals, and nanopipette size calculations.

Laser pulling process

We construct the pulled nanopipette pores as follows: We first clean 7.5 cm quartz capillaries (O.D. 1.0 mm, I.D. 0.50 mm) with an internal filament (Sutter instruments) by sonicating them upright in acetone for 15 minutes. They are then dried with compressed air and baked in a 70 °C oven for 20 min. The pulling process uses a Sutter P-2000G laser–assisted pipette puller set to following parameters: HEAT = 575, FIL = 0, VEL = 25, DEL = 180, and PUL = 225. After pulling, we secure the nanopipettes horizontally and fill them with 4 M LiCl 10 mM TE buffer using 20 μL microloader pipette tips (Eppendorf). If an air bubble was visible near the nanopipette tip, then we refill the nanopipette in the same manner until the air bubble is removed.

Sample validation

We prepare DNA solutions such that, after irradiation and dilution, the concentration of total nucleic acid is equivalent 3 nM (4.875 ng/μL) 2.5 kbp DNA. We confirm this after irradiation with UV-vis spectroscopy, Fig. S1.

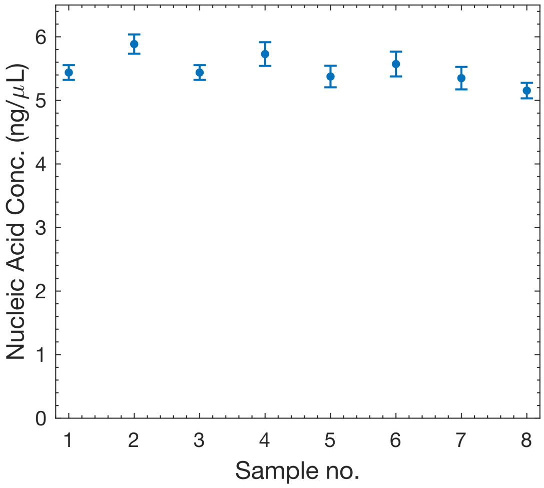

Figure S1: **Post–irradiation DNA concentration.** The DNA concentration for each of the eight irradiated samples (at doses from 0 Gy to 15 Gy). We obtain the concentration with UV light absorbance using the NanoDrop One (Thermo Fisher). Error bars show plus/minus one standard deviation over 3 runs. The concentration is essentially constant within the measurement error.

Pipette characterization

We calculate the mean and standard deviation of the nanopipette terminal diameter from the resistance of five representative nanopipettes prepared in the identical manner to those used in our primary experiments. The resistance was measured by scanning through applied voltages between -500 mV and +500 mV and noting the current as shown in Fig. S2a. These pipettes had a resistance of (47.5 $\pm$ 8.6) MΩ. The terminal diameter of (17 $\pm$ 3) nm is calculated from the resistance via the equation derived by Bell and Keyser[S1] using a value of 18.66 S/m for the conductivity of 4M LiCl. Fig. S2b shows a scanning electron microscope image of the the end of a single nanopipette prepared in the same way. The terminal diameter of this nanopipette was imaged to be 15 nm, which agrees reasonably well with the value calculated via the conductance equation.

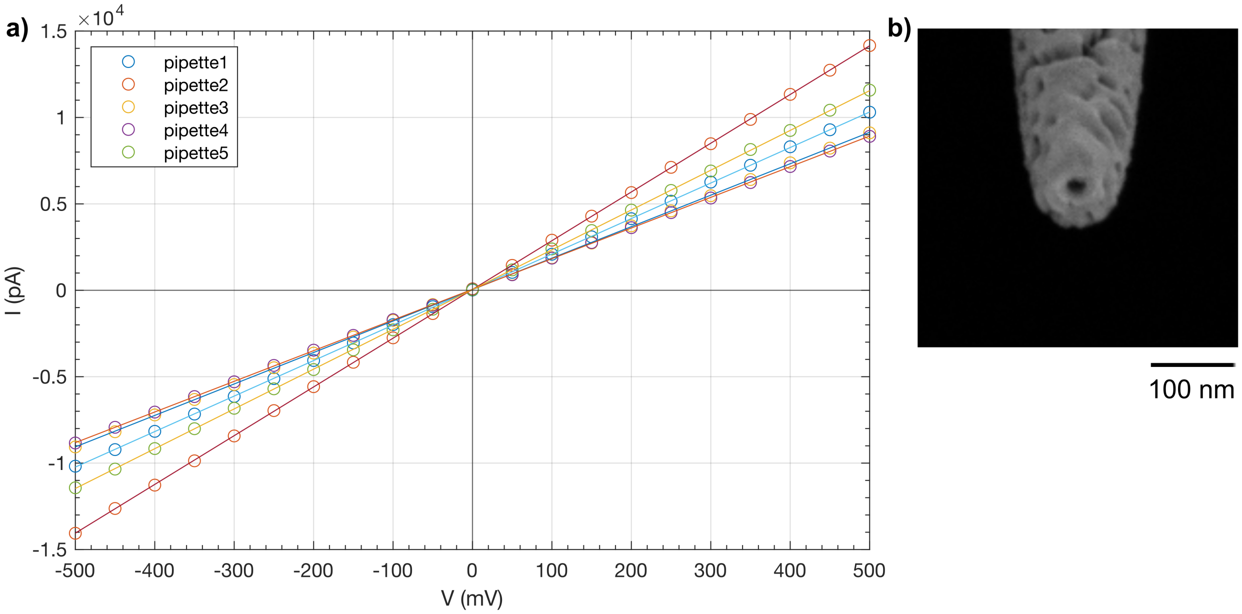

Figure S2: **Size and conductivity of pulled-quartz nanopipette.** **a)** The IV curve of five 4M LiCl filled nanopipettes prepared identically to those in the primary experiments. **b)** SEM image of the terminal end of a single pulled-quartz nanopipette prepared identically to those in the primary experiments.

Ionic current signals

Figure S3 shows the raw ionic current data (left panels) from each of the eight runs used to measure the intact DNA concentration as a function of dose, as well as direct uncorrected histograms (right panels). Each run begins with identically–prepared DNA solution from a single stock solution. As we stress in the main text, we collect each time–series with a unique nanopipette both to prevent cross contamination and to prevent fouling. We then use the Nanolyzer software package to extract and tabulate events (based on ECD). This data highlights a variety of different capture rates that appear uncorrelated to dose, as well as the variability in pore characteristics.

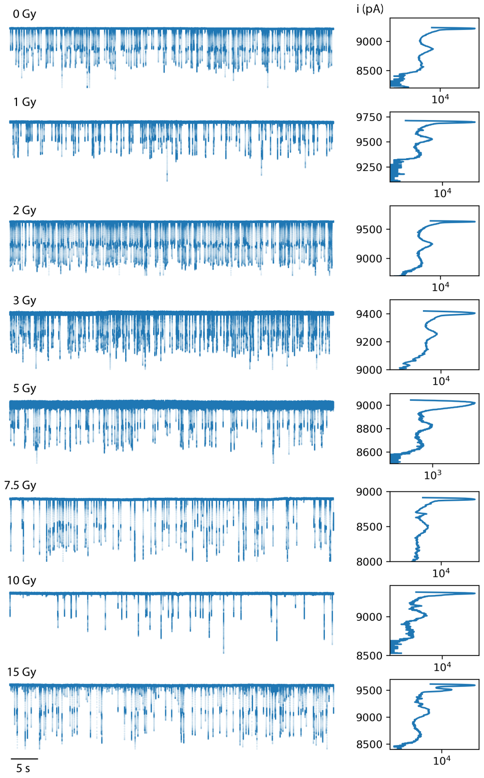

Figure S3: **Ionic current time series.** From top to bottom, the left panels show the ionic current versus time for each of the doses. The right panels show the corresponding histogram for each of these real–time traces. This data highlights the variability of individual capillaries, from the baseline current to the positions of the different peaks. The dual internal molecular standards of this work correct for this variability and enable quantitative detection of nucleic acid analytes.

References

(S1) Bell, N. A. W.; Keyser, U. F. Specific Protein Detection Using Designed DNA Carriers and Nanopores. *J. Am. Chem. Soc.* **2015**, *137*, 2035–2041.
